# Old age at diagnosis increases risk of tumor progression in nasopharyngeal cancer

**DOI:** 10.18632/oncotarget.10818

**Published:** 2016-07-24

**Authors:** Jing-Dun Xie, Fu Chen, Yao-Xuan He, Xiao-Di Chen, Guo-Ye Zhang, Zhi-Kun Li, Jing Hong, Dan Xie, Mu-Yan Cai

**Affiliations:** ^1^ Department of Anesthesiology, Sun Yat-sen University Cancer Center, State Key Laboratory of Oncology in South China, Collaborative Innovation Center for Cancer Medicine, Guangzhou, China; ^2^ Department of Clinical Laboratory, Guangdong Provincial Hospital of Traditional Chinese Medicine, Guangzhou, China; ^3^ Department of Pathology, Sun Yat-sen University Cancer Center, State Key Laboratory of Oncology in South China, Collaborative Innovation Center for Cancer Medicine, Guangzhou, China

**Keywords:** nasopharyngeal cancer, age, tumor progression, prognosis

## Abstract

Age at diagnosis has been found to be a prognostic factor of outcomes in various cancers. However, the effect of age at diagnosis on nasopharyngeal cancer (NPC) progression has not been explored. We retrospectively evaluated the relationship between age and disease progression in 3,153 NPC patients who underwent radiotherapy, chemotherapy, or chemoradiotherapy between 2007 and 2009. Patients were randomly assigned to either a testing cohort or a validation cohort by computer-generated random assignment. X-tile plots determined the optimal cut-point of age based on survival status to be ≤61 *vs*. >61 years. Further correlation analysis showed that age >61 years was significantly correlated with the tumor progression and therapeutic regimen in both testing and validation cohorts (*P* <0.05). In the present study, we observed that older age (>61 years) was a strong and independent predictor of poor disease-free survival (DFS) and cancer-specific survival (CSS), in both univariate and multivariate analyses. Age was also found to be a significant prognostic predictor as well (*P* <0.05) when evaluating patients with the same disease stage. ROC analysis confirmed the predictive value of age on NPC-specific survival in both cohorts (*P* <0.001) and suggested that age may improve the ability to discriminate outcomes in NPCs, especially regarding tumor progression. In conclusion, our study suggests that older age at NPC diagnosis is associated with a higher incidence of tumor progression and cancer-specific mortality. Age is a strong and independent predictor of poor outcomes and may allow for more tailored therapeutic decision-making and individualized patient counseling.

## INTRODUCTION

Nasopharyngeal cancer (NPC) is a malignant tumor that arises from the epithelial surface of the posterior wall of the nasopharynx [1]. NPC differs from other head and neck cancers because of its striking ethnic and geographic distribution, disproportionately affecting certain areas of Asia. According to the International Agency for Research on Cancer, the average annual incidence of NPC worldwide is less than 1/100,000 people; however, in endemic areas of China, the annual incidence reaches 20/100,000 people [2-5]. Most NPCs are undifferentiated with a tendency to be locally invasive and metastasize to neck lymph nodes. Early-stage NPC is highly radiocurable, but local treatment failure and distant metastasis are still major causes of adverse outcomes in patients with advanced stage NPC. Conventional TNM staging is a strong prognostic indicator in NPC, but few other clinical variables have been identified as good predictors of tumor progression.

Age at diagnosis is a prognostic factor of cancer-specific survival (CSS) for various cancers [6-12]. Identification of age as a prognostic factor helps clinicians utilize the most appropriate treatment strategies for cancer patients at different ages. A few studies have demonstrated that age at diagnosis is associated with survival in patients with NPC; however, these results come from studies with a small number of cases and there is a lack of independent validation of the results. Additionally, the selection of the age cut-off point used in these studies was arbitrary. More importantly, these studies did not assess the effect of age at diagnosis on progression of NPC. Statistical analysis could be used to select the optimal age cut-point that could best identify patients as high or low risk for cancer-specific mortality. These results would be valuable for treatment selection and patient counseling.

In the present study, we constructed two independent NPC cohorts from our institution. We used the X-tile program, a bioinformatics software tool for cut-point optimization [13], to determine the optimal age cutoff in the first cohort of NPC patients to test the effect of age at diagnosis on tumor progression in NPCs. These findings were confirmed and validated in the second cohort. In the present study, we found that age > 61 correlates closely with tumor progression and poor prognosis in patients with NPC and maximizes the predictive value of age on a patient's survival.

## RESULTS

### Patient characteristics

Patients were assigned to a testing cohort or a validation cohort by computer-generated random number assignment. The testing cohort (*n* = 1,577) included 1,204 (76.3%) men and 373 (23.7%) women with a median age of 46 years. Among 1,577 NPC patients in the testing cohort, regional recurrence and distant metastasis were observed in 115 (7.3%) and 129 (8.2%) patients, respectively. Average follow-up time was 53.96 months (median, 58.28 months; range, 5.01-91.63 months). The validation cohort (*n* = 1,576) consisted of 1,150 (73.0%) men and 426 (27.0%) women with a median age of 45 years. Among 1,576 patients in the validation cohort, regional recurrence and distant metastasis were detected in 109 (6.9%) and 142 (9.0%) patients, respectively. Average follow-up time was 53.34 months (median, 56.68 months; range, 5.03-103.45 months).

### Relationship between age at diagnosis and clinicopathologic features

**Figure 1:**
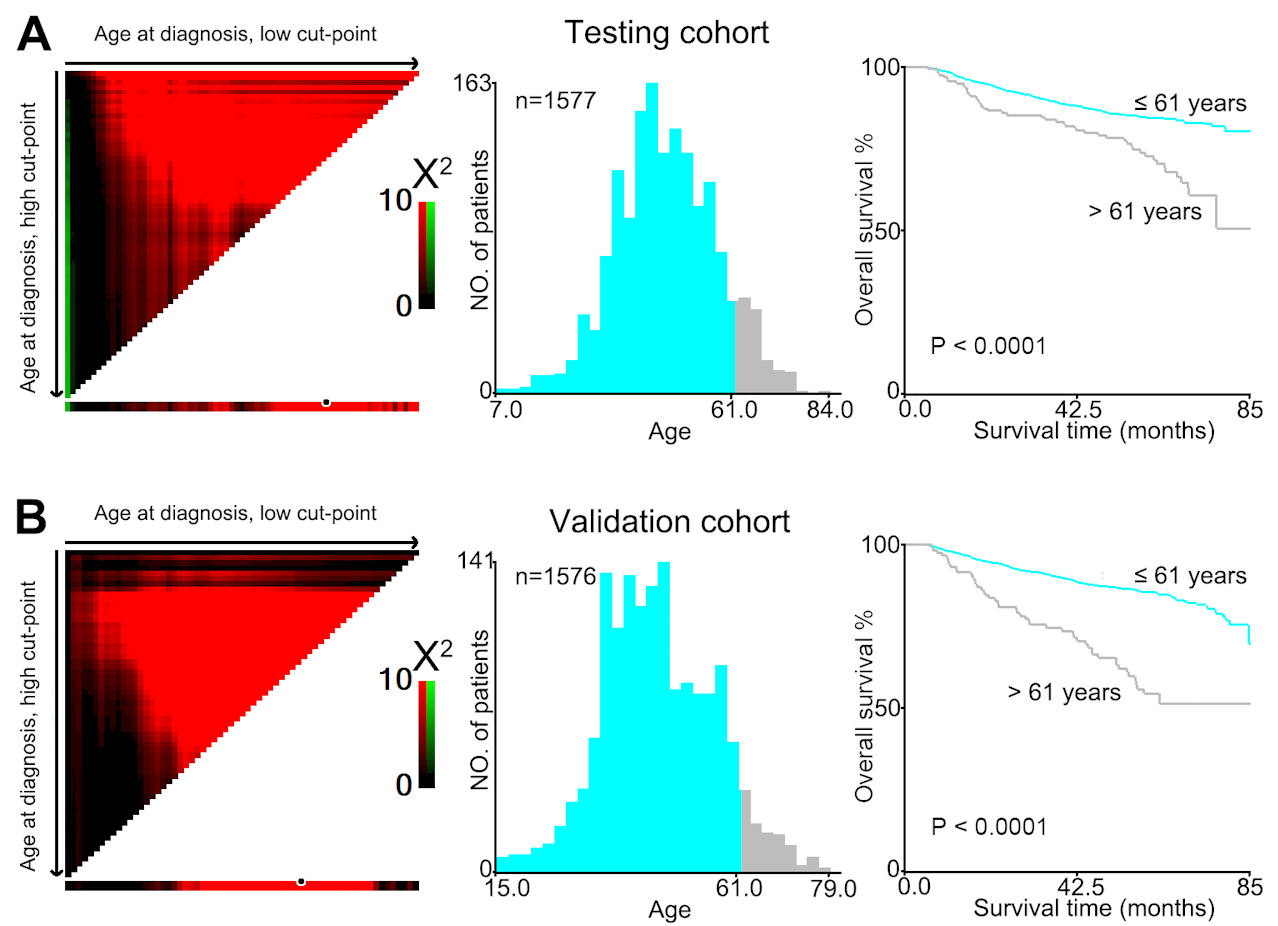
X-tile plots were utilized to determine the cutoff value of the age at diagnosis on NPC cohorts The X-tile program analyzed patient data from the testing cohort. The cutpoint highlighted by the black/white circle in the left panels was demonstrated on a histogram of the entire cohort (middle panels) and a Kaplan-Meier plot (right panels). **A.** Age at diagnosis was divided at the optional cutpoint, as defined by the most significant on the plot (≦ 61 years *vs* > 61 years; *P* < 0.0001). **B.** The optional cutpoint for age at diagnosis determined by the X-tile plot of the testing cohort was applied to the validation cohort and remained statistically significant (*P* < 0.0001).

The X-tile program determined cutoff scores for age. According to the X-tile plots, a cutpoint of 61 years most appropriately divided the testing cohort into young and old populations (*P* < 0.0001, Figure 1A). This optimal cutpoint was applied to the validation cohort and was found to again be highly statistically significant (*P* < 0.0001, Figure 1B). In the testing cohort, there were 163 NPC patients > 61 years (10.4%). Further correlation analysis showed that therapeutic regimens and disease progression were significantly different between the young and old populations (*P* < 0.05, Table 1).

In the validation cohort, there were 119 NPC patients > 61 years (7.6%). Similarly, correlation analysis revealed that N stage, M stage, therapeutic regimen and disease progression were significantly different between the young and old populations (*P* < 0.05, Table 1).

**Table 1:** The relationship of Age with patient's clinicopathological features in primary nasopharyngeal cancer

	Age							
Variable	Testing cohort				Validation cohort			
	All cases	≤ 61	> 61	*P* value^*^	All cases	≤ 61	> 61	*P* value^*^
Sex				0.202				0.972
Male	1204	1073 (89.1%)	131(10.9%)		1150	1063(92.4%)	87 (7.6%)	
Female	373	341 (91.4%)	32 (8.6%)		426	394 (92.5%)	32 (7.5%)	
Histological classification (WHO)				0.286				0.385
Type II	165	144 (87.8%)	21 (12.7%)		162	143 (88.3%)	19(11.7%)	
Type III	1412	1270 (89.9%)	142(10.1%)		1414	1310(92.6%)	104(7.4%)	
T stage				0.169				0.123
1	100	94 (94.0%)	6 (6.0%)		94	91 (96.8%)	3 (3.2%)	
2	356	324(91.0%)	32 (9.0%)		361	336 (93.1%)	25 (6.9%)	
3	735	659 (89.7%)	76 (10.3%)		758	703 (92.7%)	66 (7.3%)	
4	386	337 (87.3%)	49 (12.7%)		363	327 (90.1%)	36 (9.9%)	
N stage				0.457				0.034
0	287	250 (87.1%)	37 (12.9%)		309	275 (89.0%)	34(11.0%)	
1	603	546 (90.5%)	57 (9.5%)		582	543 (93.3%)	39 (6.7%)	
2	555	499 (89.9%)	56 (10.1%)		551	518 (94.0%)	42 (6.0%)	
3	132	119 (90.2%)	13 (9.8%)		134	121 (90.3%)	13 (9.7%)	
M stage				0.356				0.001
No	1495	1338 (89.5%)	157(10.5%)		1509	1402(92.9%)	107(7.1%)	
Yes	82	76 (92.7%)	6 (7.3%)		67	55 (82.1%)	12(17.9%)	
Therapeutic regimen				0.000				0.000
RT	268	219 (81.7%)	49 (18.3%)		265	231 (87.2%)	34(12.8%)	
CT	99	91(91.9%)	8(8.1%)		112	99 (88.4%)	13(11.6%)	
CRT	1210	1104 (91.2%)	106 (8.8%)		1199	1127(94.0%)	72 (6.0%)	
Progression				0.000				0.000
No	1318	1203 (91.3%)	115 (8.7%)		1307	1238(94.7%)	69 (5.3%)	
Yes	259	211 (81.5%)	48 (18.5%)		269	219 (81.4%)	50(18.6%)	

* Chi-square test; WHO, World Health Organization; RT, radiotherapy; CT, chemotherapy; CRT, chemoradiotherapy.

### Association between age at diagnosis of NPC and patient survival: univariate survival analysis

**Figure 2:**
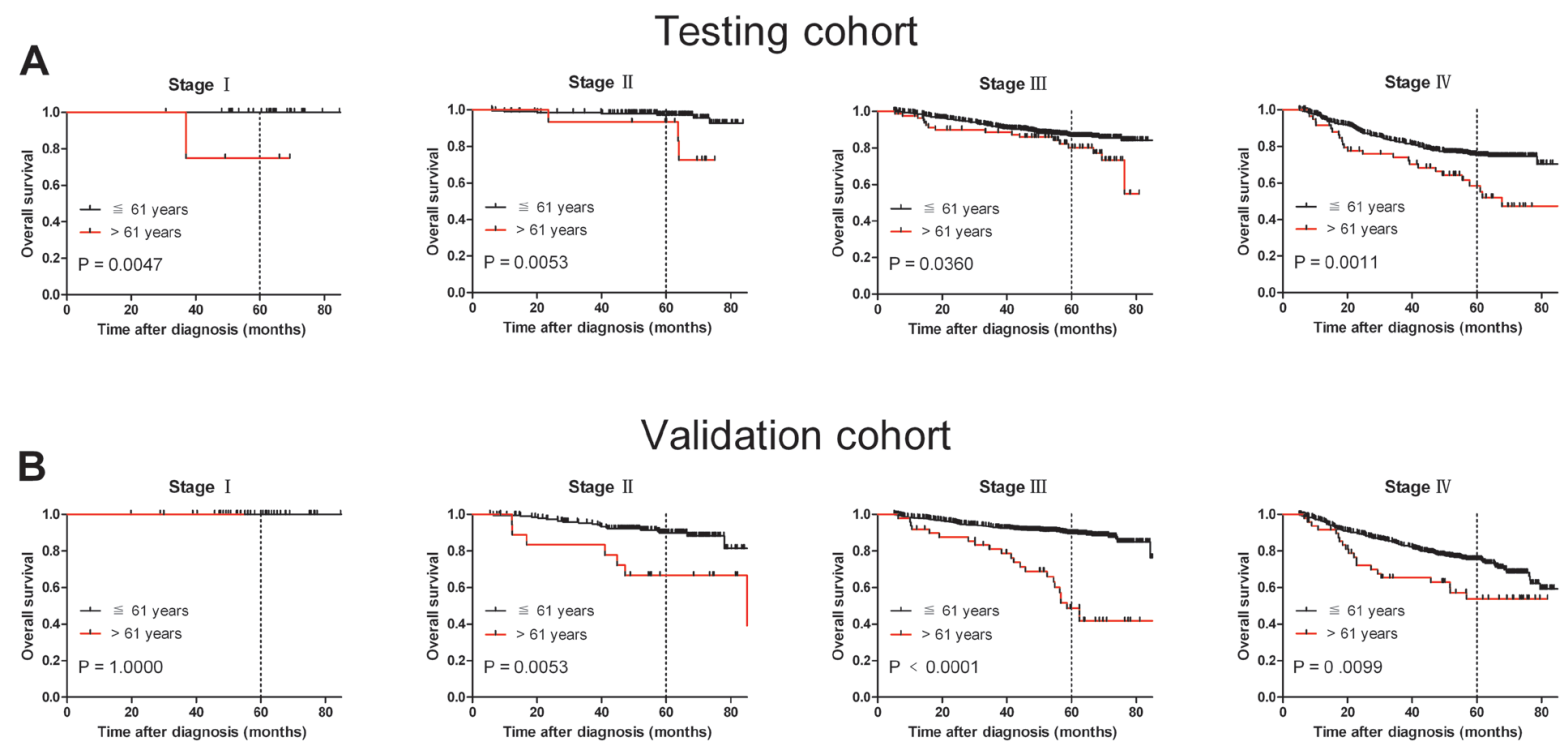
Age at diagnosis was correlated NPC patients' shorter overall survival in subsets of different tumor stages (log-rank test) **A.** Stage I, probability of survival of stage I patients with NPC in the testing cohort; ≦ 61 years, *n* = 33; > 61 years, *n* = 4. Stage II, probability of survival of stage II patients with NPC in the testing cohort; ≦ 61 years, *n* = 211; > 61 years, *n* = 15. Stage III, probability of survival of stage III patients with NPC in the testing cohort; ≦ 61 years, *n* = 697; > 61 years, *n* = 82. Stage IV, probability of survival of stage IV patients with NPC in the testing cohort; ≦ 61 years, *n* = 473; > 61 years, *n* = 62. **B.** Stage I, probability of survival of stage I patients with NPC in the validation cohort; ≦ 61 years, *n* = 42; > 61 years, *n* = 2. Stage II, probability of survival of stage II patients with NPC in the validation cohort; ≦ 61 years, *n* = 206; > 61 years, *n* = 19. Stage III, probability of survival of stage III patients with NPC in the validation cohort; ≦ 61 years, *n* = 758; > 61 years, *n* = 49. Stage IV, probability of survival of stage IV patients with NPC in the validation cohort; ≦ 61 years, *n* = 451; > 61 years, *n* = 49.

To confirm the representativeness of the cases in the testing cohort, we first tested well-established prognostic factors of patient survival in those with NPC. Kaplan-Meier analysis was used to evaluate well-known clinical pathological prognostic parameters on patients' survival, including sex (*P* < 0.05), T stage, N stage, M stage, therapeutic regimen and age (*P* < 0.0001, Table 2). This analysis revealed that age > 61 year was associated with adverse overall survival (OS) (*P* < 0.0001, Figure 1A and Table 2). Further analysis was performed with patients stratified according to clinical stage; results of this sub-analysis demonstrated that age > 61 years was also an adverse prognostic factor of OS within cohorts grouped by stage: stage I (*P* = 0.0047), stage II (*P* = 0.0053), stage III (*P* = 0.036) and stage IV (*P* = 0.0011, Figure 2A).

**Table 2:** Univariate analysis of different prognostic variables in 3153 patients with nasopharyngeal carcinoma

Variable	Testing cohort				Validation cohort			
	All cases	Mean survival (months)	Median survival (months)	*P* value^*^	All cases	Mean survival (months)	Median survival (months)	*P* value^*^
Sex				0.018				0.003
Male	1204	78.0	88.6		1150	82.8	89.2	
Female	373	79.0	NR		426	78.7	NR	
Histological classification (WHO)				0.354				0.543
Type II	165	71.8	NR		162	74.8	NR	
Type III	1412	79.1	88.6		1414	83.8	89.2	
T stage				0.000				0.000
1	100	85.1	NR		94	78.9	NR	
2	356	81.5	88.6		361	79.8	89.2	
3	735	76.7	86.		758	86.8	NR	
4	386	74.5	NR		363	69.8	NR	
N stage				0.000				0.000
0	287	81.3	NR		309	81.5	89.2	
1	603	78.5	NR		582	92.2	NR	
2	555	74.0	86.7		551	78.0	NR	
3	132	75.1	NR		134	58.2	69.3	
M stage				0.000				0.000
0	1495	79.9	88.6		1509	85.0	89.2	
1	82	54.6	63.6		67	46.3	40.4	
Therapeutic regimen				0.000				0.000
RT	268	80.9	NR		265	87.6	89.2	
CT	99	67.7	NR		112	62.5	76.6	
CRT	1210	78.4	88.6		1199	79.6	NR	
Age				0.000				0.000
≤ 61	1414	78.8	88.6		1457	87.7	NR	
> 61	163	70.9	NR		119	62.6	89.2	

* log-rank test; WHO, World Health Organization; RT, radiotherapy; CT, chemotherapy; CRT, chemoradiotherapy.

Results in the validation cohort were similar to those in the testing cohort. Patients > 61 years had worse OS compared with patients ≦ 61 years (*P* < 0.0001; Figure 1B and Table 2). Univariate analysis demonstrated that sex (*P* < 0.05), T stage, N stage, M stage, therapeutic regimen and age (*P* < 0.0001, Table 2) adversely affected overall patient survival. In addition, survival analysis stratified by stage showed that age > 61 years was a prognostic predictor in stage II (*P* = 0.0053), stage III (*P* < 0.0001) and stage IV (*P* = 0.0099, Figure 2B).

**Figure 3:**
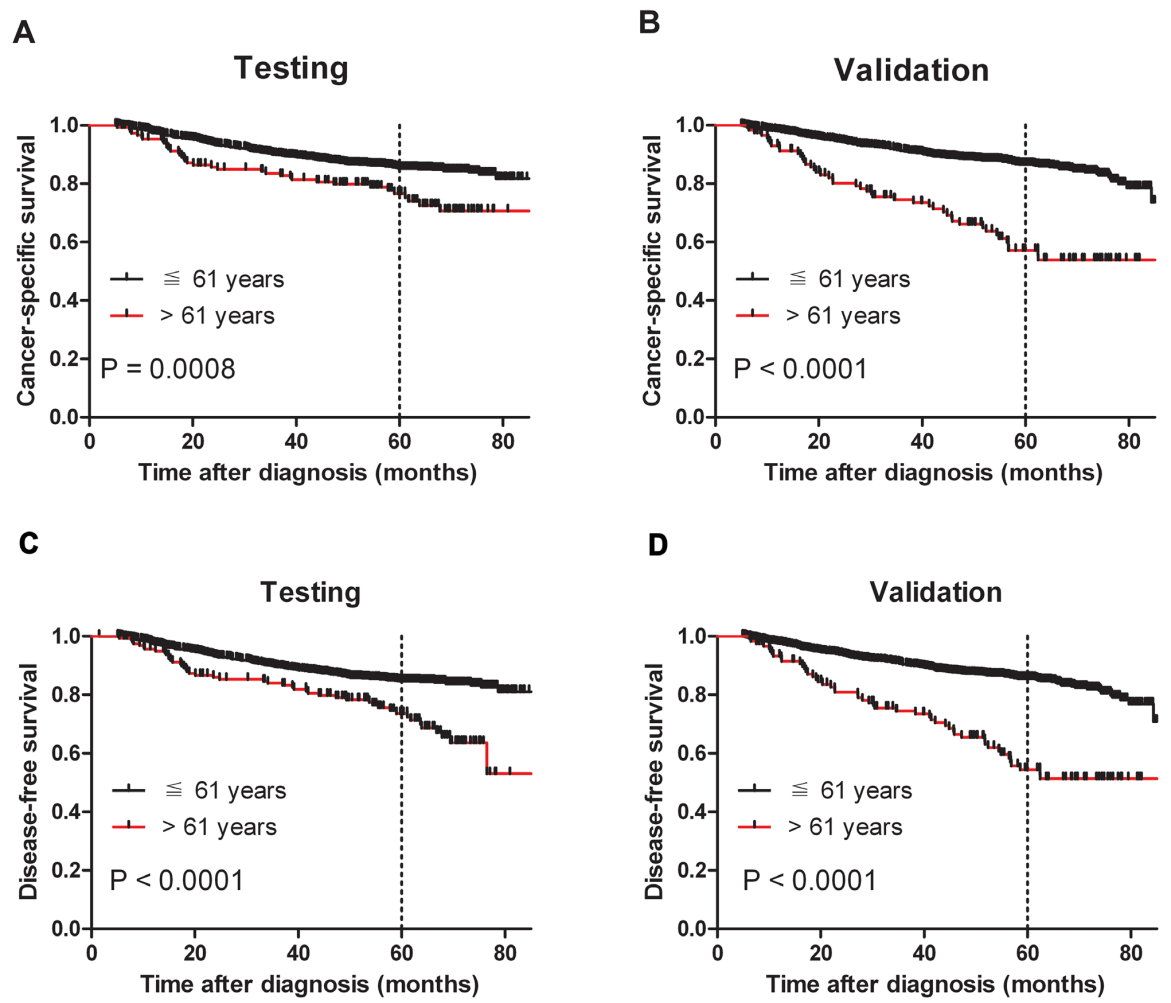
Older age was associated with shorter NPC cancer-specific survival and disease-free survival (log-rank test) **A.** Probability of cancer-specific survival of patients with NPC in the testing cohort; ≦ 61 years, *n* = 1404; > 61 years, n = 153. **B.** Probability of cancer-specific survival of patients with NPC in the validation cohort; ≦ 61 years, *n* = 1435; > 61 years, *n* = 115. **C.** Probability of disease-free survival of patients with NPC in the testing cohort; ≦ 61 years, *n* = 1414; > 61 years, *n* = 163. **D.** Probability of disease-free survival of patients with NPC in the validation cohort; ≦ 61 years, *n* = 1457; > 61 years, *n* = 119.

**Figure 4:**
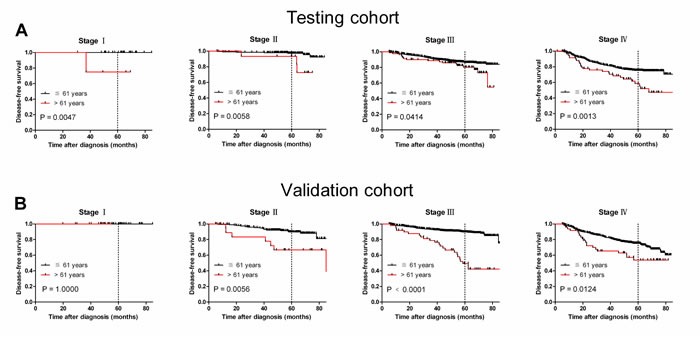
Older age was associated with shorter NPC disease-free survival in subsets of different cancer stages (log-rank test) **A.** Stage I, probability of survival of stage I patients with NPC in the testing cohort; ≦ 61 years, *n* = 33; > 61 years, *n* = 4. Stage II, probability of survival of stage II patients with NPC in the testing cohort; ≦ 61 years, *n* = 211; > 61 years, *n* = 15. Stage III, probability of survival of stage III patients with NPC in the testing cohort; ≦ 61 years, *n* = 697; > 61 years, *n* = 82. Stage IV, probability of survival of stage IV patients with NPC in the testing cohort; ≦ 61 years, *n* = 473; > 61 years, *n* = 62. **B.** Stage I, probability of survival of stage I patients with NPC in the validation cohort; ≦ 61 years, *n* = 42; > 61 years, *n* = 2. Stage II, probability of survival of stage II patients with NPC in the validation cohort; ≦ 61 years, *n* = 206; > 61 years, *n* = 19. Stage III, probability of survival of stage III patients with NPC in the validation cohort; ≦ 61 years, *n* = 758; > 61 years, *n* = 49. Stage IV, probability of survival of stage IV patients with NPC in the validation cohort; ≦ 61 years, *n* = 451; > 61 years, *n* = 49.

**Figure 5:**
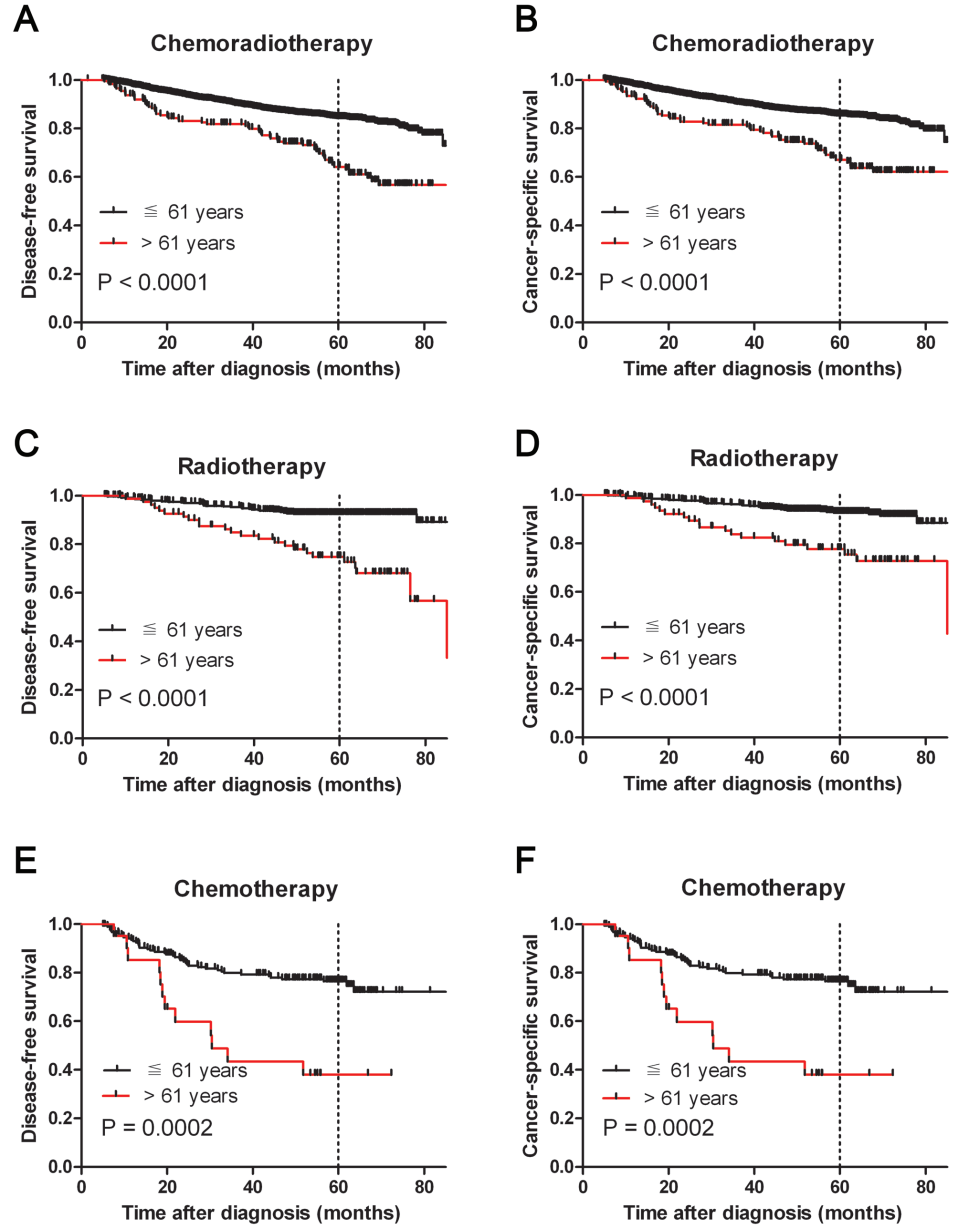
Kaplan-Meier survival analysis of age at diagnosis in subsets of total NPC patients in different therapeutic regimen groups (log-rank test) Probability of survival of NPC patients with chemoradiotherapy in disease-free survival **A.** and cancer-specific survival **B.** Probability of survival of NPC patients with radiotherapy in disease-free survival **C.** and cancer-specific survival **D.** Probability of survival of NPC patients with chemotherapy in disease-free survival **E.** and cancer-specific survival **F.**

Our results suggest that age > 61 years is an unfavorable predictor for overall survival in NPC patients. To further ascertain whether this effect was related to NPC progression, we examined the association between age > 61 years with cancer-specific survival (CSS) and disease-free survival (DFS), respectively. Our data confirmed that age > 61 years was associated with adverse CSS and DFS in both cohorts (*P* < 0.05, Figure 3A, 3B, 3C and 3D). Assessment of DFS in the testing cohort revealed that age > 61 years was an adverse prognostic factor for NPC patients when stratifying on stage: stage I (*P* = 0.0047), stage II (*P* = 0.0058), stage III (*P* = 0.0414) and stage IV (*P* = 0.0013, Figure 4A). The validation cohort also supported the finding that age > 61 years carries a worse prognosis regardless of stage for stage II (*P* = 0.0056), stage III (*P* < 0.0001) and stage IV (*P* = 0.0124, Figure 4B). In addition, therapy-regimen-match survival analysis showed that age > 61 years was a prognostic predictor for NPC patients either in chemoradiotherapy, radiotherapy or chemotherapy subgroups in total patients (*P <* 0.001 for all, Figure 5).

### Association between age at NPC diagnosis and patient survival: multivariate Cox regression analysis

**Figure 6:**
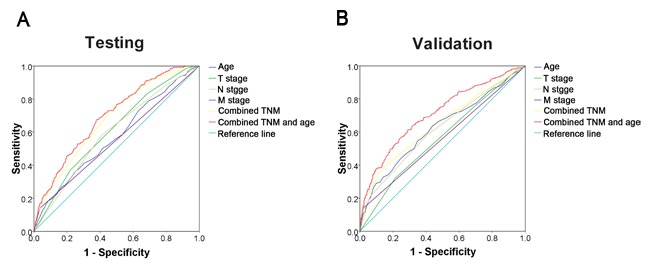
ROC curve analysis for different clinicopathological features was performed to evaluate the survival status **A.** Age (AUC = 0.573; *P* < 0.001), T stage (AUC = 0.613; *P* < 0.001), N stage (AUC = 0.595; *P* < 0.001), M stage (AUC = 0.552; *P* = 0.008), combined TNM (AUC = 0.678; *P* < 0.001), and combined TNM and age (AUC = 0.696; *P* < 0.001) indicated significant associations with survival status in the testing cohort. **B.** Age (AUC = 0.623; *P* < 0.001), T stage (AUC = 0.564; *P* = 0.001), N stage (AUC = 0.616; *P* < 0.001), M stage (AUC = 0.560; *P* = 0.002), combined TNM (AUC = 0.650; *P* < 0.001), and combined TNM and age (AUC = 0.709; *P* < 0.001) were used to test the survival status in the validation cohort.

Because some prognostic variables in the univariate analysis may covariate, we performed a multivariate analysis of age and other clinicopathological parameters that were significant in both cohorts in the univariate analysis (Table 3). In the testing cohort, age > 61 years was found to be a significant independent prognostic factor for poor CSS (hazard ratio, 2.085; 95% confidence interval [CI], 1.505-2.889, *P* < 0.0001; Table 3). Similar results were also observed in the validation cohort (hazard ratio, 2.896; 95% confidence interval [CI], 2.106-3.983, *P* < 0.0001; Table 3). We also evaluated sex, T stage, N stage, and M stage as independent prognostic factors for patient survival in both cohorts by plotting ROC curves to test patient survival status. ROC curve analysis confirmed the predictive value of age regarding NPC-specific survival in the testing cohort (area under the curve, AUC = 0.548, Figure 6A) and was supported in the validation cohort (AUC = 0.568, Figure 6B). Applying Harrell's C-index to test the predictive ability of integrating age into the clinicopathologic model in NPC patients, we found that age improved the predictive ability when compared with the clinicopathologic model alone (C-indexes from 0.678 to 0.696 and from 0.650 to 0.709, respectively).

**Table 3:** Cox Multivariate analyses of prognostic factors on cancer-specific survival

Testing cohort			
Characteristics	HR	HR(95% CI)	*P* value
Sex (female *vs* male)	1.480	1.480 (1.070- 2.046)	**0.018**
Histological classification (WHO) (type II *vs* type III)	0.846	0.846 (0.579- 1.236)	0.387
T stage (1 *vs* 2 *vs* 3 *vs* 4)	1.641	1.641 (1.386-1.943)	**0.000**
N stage (0 *vs* 1 *vs* 2 *vs* 3)	1.448	1.448 (1.250-1.677)	**0.000**
M stage (no *vs* yes)	3.479	3.479 (2.368-5.109)	**0.000**
Therapeutic regimen (RT *vs* CT *vs* RCT)	1.063	1.063 (0.863-1.309)	0.566
Age, years (≤61 *vs* >61)	2.085	2.085 (1.505-2.889)	**0.000**
**Validation cohort**			
**Characteristics**	**HR**	**HR(95% CI)**	***P* value**
Sex (female *vs* male)	1.527	1.527 (1.127-2.047)	**0.006**
Histological classification (WHO) (type II *vs* type III)	0.991	0.991 (0.681-1.440)	0.961
T stage (1 *vs* 2 *vs* 3 *vs* 4)	1.324	1.324 (1.130-1.552)	**0.001**
N stage (0 *vs* 1 *vs* 2 *vs* 3)	1.572	1.572 (1.364-1.811)	**0.000**
M stage (no *vs* yes)	4.051	4.051 (2.741-5.989)	**0.000**
Therapeutic regimen (RT *vs* CT *vs* RCT)	0.988	0.998 (0.811-1.205)	0.908
Age, years (≤61 *vs* >61)	2.896	2.896 (2.106-3.983)	**0.000**

CI, confidence interval, WHO, World Health Organization; RT, radiotherapy; CT, chemotherapy; CRT, chemoradiotherapy.

## DISCUSSION

Many clinicopathologic features have been associated with poor CSS from NPC [14, 15]. However, the role of age and the degree to which age affects NPC progression is unclear. When a continuous variable is demonstrated to be a prognostic factor in disease progression, the selection of an optimal cutpoint can maximize its predictive value and best separate patients with high risk of death from cancer due to tumor progression from patients with low risk. We investigated the effect of age on NPC disease progression using two independent groups of NPC patients.

To assess the prognostic significance of age and to avoid arbitrary predetermined cutpoints, we constructed X-tile plots to assess age using two groups and found age ≦ 61 years versus > 61 years to be the most effective cutpoint once corrected for the use of minimum P statistics by Miller-Siegmund P-value correction [13]. Further correlation analysis revealed that age at diagnosis was closely associated with the progression and survival of NPC patients. There is no general consensus on the influence of age at diagnosis on survival in NPC [16, 17]. Since many factors can contribute to adverse survival outcomes in older individuals, for example cardiovascular and respiratory diseases, we excluded non-NPC related deaths and found that age > 61 years remained a prognostic factor of not only OS from NPC but also of CCS. Our results are consistent with previous research [15, 18]. Since the majority of mortality from NPC results from tumor progression, including both tumor recurrence and distant metastasis, the effect of age on DFS at different clinical stages of both cohorts was assessed. There was a significant difference in DFS between patients ≦ 61 years and > 61 years in each subset of clinical stage. Our data suggested that older age is an independent adverse prognostic factor for tumor progression and survival in NPC.

Multivariate analyses in both cohorts revealed that older age at NPC diagnosis was a prognostic parameter independent of other well-established clinical factors, including sex, WHO classification, TNM stages and therapeutic regimen. Similarly, previous studies have reported that age was a prognostic factor for poor outcomes from NPC as evidenced by multivariate analysis [19, 20]. Older patients are more likely to have comorbidities and poorer performance status, which may render older patients unable to tolerate certain cancer treatments. Older patients with severe comorbidities exhibited low completion rates of standard treatment. We hypothesize that poorer performance status may contribute to the lower survival rate in older NPC patients. Our findings, combined with those of previous studies, provide evidence that older age at diagnosis may be a significant independent prognostic factor in NPC [21-23]. Therefore, age and age-related performance status should be considered when treating a patient for NPC.

Currently, the tumor-node-metastasis (TNM) staging system remains the major tool for developing treatment strategies and evaluating clinical prognosis in cancer. However, there may be wide variability in the clinical outcomes observed among patients with the same tumor stage receiving similar treatment [24]. This suggests that the current TNM staging system alone might be inadequate for therapeutic decision-making and prognostic prediction of certain cancers [25, 26]. Thus, there is a need for additional objective strategies that can further effectively distinguish between patients with favorable and unfavorable outcomes. In the present study using two large cohorts of NPC patients, we observed that older age was a strong and independent predictor of short cancer specific survival, as evidenced by Kaplan-Meier curves and multivariate Cox proportional hazards regression analysis. Moreover, ROC curve analysis supports the idea that an older age at diagnosis may improve the ability to discriminate between prognostic outcomes for NPC patients, especially in regards to tumor progression. Our data support the concept that taking age at diagnosis into consideration can help identify NPC patients at risk for an aggressive clinical course and/or poor outcome.

This study has several limitations. The analysis is retrospective in nature. Secondly, as all patients included in this study came from China, generalizability to other geographic regions is limited. Further studies from other geographical areas are required to validate and generalize our results. The possible confounders for existing co-morbidities among older patients may also play a role in terms of limiting them from completing the prescribed treatment, especially concurrent chemo-radiation which is known to have toxicity related issues among older patients. Smoking is a risk factor for NPC; the rate of smoking in males is higher than in females in China, but we did not take this into consideration. Furthermore, the underlying causes of the gender differences in behavior of NPC are not completely understood.

In summary, our study confirmed that older age at NPC diagnosis is associated with a higher incidence of tumor progression and cancer-specific mortality and is a strong and independent predictor of a poor outcome, as indicated by univariate and multivariate analyses. The addition of age into the TNM model could improve the ability to prognosticate outcomes for patients with NPC. Our data suggest that age can function as an independent prognostic factor of outcomes in NPC and support the consideration of considering age at diagnosis of primary NPC to facilitate therapeutic decisions and individualized patient counseling.

## MATERIALS AND METHODS

### Patients and cohorts

We identified 3,153 patients from the database at the Sun Yat-sen University Cancer Center who underwent radiotherapy (RT), chemotherapy (CT), or chemoradiotherapy (CRT) for NPC between 2007 and 2009. Case selection was based on the following criteria: pathologically confirmed nonkeratinizing carcinoma of the nasopharynx (World Health Organization types of II or III); no previous malignancy or second primary tumor; no previous radiotherapy, chemotherapy or surgical treatment of nasopharynx before diagnosis; Karnofsky score ≥ 70; received RT, IC/RT or IC/CRT for treatment of NPC, and had regular follow-up. Clinical variables collected for each patient included age at diagnosis, gender, TNM stage, histological subtype, therapeutic regimens, and survival time. Clinicopathological features are summarized in Table 1.

Tumor stage was defined according to the American Joint Committee on Cancer/International Union Against Cancer TNM (tumor-node-metastasis) classification system [27]. The institutional research medical ethics committee of Sun Yat-sen University granted approval for this study.

### Follow-up

Patients were followed every 3 months for the first year, every 6 months for the next 2 years, and then annually thereafter. Follow-up examinations consisted of fiber optic nasopharyngoscopy, MRI, CT, chest X-ray, PET-CT, abdominal ultrasonography and bone scan when necessary to detect recurrence and/or metastasis. Overall survival time was determined from the date of diagnosis to the date of death from any cause or last follow-up. Disease-free survival was determined from the date of diagnosis to recurrence or to the date of death from any cause. Disease progression was defined as cases in which the tumor was evaluated as progressive disease (PD) after treatment for the primary tumor or recurrence after CR (local progression) and/or cases in which new distant metastasis occurred (distant progression).

### Selection of cutoff for age at diagnosis

X-tile plots were generated to assess age and to optimize the age cutpoint based on patients' survival status [13]. The X-tile program divided the cohorts randomly into matched training and validation sets in order to select the optimal cutoff. Statistical significance was assessed through using the cutoff score derived from the training set to parse a separate validation set. We used a standard log-rank method with p-values obtained from a lookup table. The X-tile plots determined an optimal cutoff value while correcting for the use of minimum P statistics by Miller-Siegmund P-value correction [28].

### Statistical analysis

Optimal cutoff for age based on survival analysis was obtained by using X-tile software version 3.6.1 (Yale University School of Medicine, New Haven, CT, USA) as described previously [13]. We used Mantel-Cox log-rank test to determine statistical significance of the correlation between age and patient survival. Monte Carlo simulations were used to adjust for multiple observations in optimal cutpoint selection [28]. Receiver operating characteristic (ROC) curve analysis was used to evaluate the predictive value of the parameters. Harrell's concordance index (C-index) assessed the model's prognostic accuracy in the multivariate analysis. Correlations between variables, ROC curve analysis, stage-match univariate survival analysis and multiple Cox proportional hazards regression were performed using SPSS statistical software package (SPSS Standard version 13.0; SPSS, Chicago, IL, USA). A two tailed p-value of < 0.05 was considered statistically significant.
